# Medical and Philosophical Intelligence

**Published:** 1819-01

**Authors:** 


### MEDICAL AND PHILOSOPHICAL INTELLIGENCE.

*PROCEEDINGS of the Royal Society.*—Amongst the proceedings of this Society which relate to Medicine, we have to notice the *Croonian Lecture*, read November the 5th, by Sir EVERARD HOME.

The subject was the conversion of pus into granulations of new flesh. The object of the author was to show that granulations, which appear to consist of a congeries of tortuous vessels, are formed in a manner very similar to the blood-vessels, &c. described by him in a former paper.* Pus, according to Sir E. Home, when first secreted, is a transparent fluid. A pellicle of pus in this state covers the little prominences of the granulations. Under this pellicle particles of air are exuded, upon which vessels appear to be moulded. These vessels soon become distended with red blood. They anastomose freely, and chiefly lie horizontally, though minute red spots are also apparent, which seem to be the termination of vessels running in a perpendicular direction. The air exuded, and upon which the vessels are formed, the author considers to be carbonic acid gas.

Drawings, illustrative of these and the subsequent changes, made chiefly from the observations of Mr. Baüer, accompanied the paper.

---

The fifty-seventh Number of the Edinburgh Medical and Surgical Journal contains a relation of two interesting cases of *Mercurial Erethismus*, by Dr. ASTBURY.

A man, aged 55, of a strong muscular appearance, but with weak stomach and bowels, consulted Dr. A. on Dec. 19, 1814, on account of a cold œdematous swelling of the right leg and thigh, the consequence of a diseased lymphatic gland in the groin. Some mild mercurial ointment, combined with camphor and soft-soap, was directed to be rubbed on the thigh of the affected side, morning and evening. On the 29th of December, Dr. A. found the patient raised up in bed, with his mouth open, gasping for breath, his breathing being very laborious. He complained of great uneasiness and anxiety about the præcordia: he was in a state of profuse perspiration; his pulse fluttering, feeble, and intermitting, so that it could not be counted; there were slight pain of the side and cough present, with trifling bloody expectoration; his tongue was white and moist; and his breath strongly tainted with mercurial fœtor. Wine was given freely, and his bowels purged with sulphate of magnesia. The next morning his pulse was more distinct, though still intermitting; his breathing was still laborious, and the perspiration profuse, but the anxiety about the heart was diminished. The wine and sulphate of magnesia were continued

* See *London Medical and Physical Journal*, vol. xxxix. p. 253.

for several days. As the effects of the mercury declined, the pulse became more full and regular, the perspiration diminished, and the strength improved; but the bloody expectoration and cough, with slight pain of the head, increased. The wine was then omitted; and saline medicines, with small doses of nitre, and a vesicatory to the side, were directed. The swelling of the leg and thigh, and the diseased gland, gradually subsided, and the patient at length recovered his usual health.

On the 1st of January, 1815, Dr. A. was consulted on a case, where the patient had been rubbing in mercury for a venereal complaint, for some weeks. He stood in the street in a current of air on December 31, 1814, in thin shoes, for a considerable time, and was attacked in the evening with great anxiety about the precordia; alarming and sudden depression of strength; numbness and partial loss of motion of his hands and feet; and great agitation of mind. He was in a state of profuse perspiration; his pulse was fluttering and undulating, and his tongue dry. On the following morning, Dr. A. found him in a state of profuse perspiration; his pulse full and strong, beating about sixty in a minute, but very intermitting; his breathing tolerably free; his tongue was dry, and brown in the middle; his countenance composed, and his mind clear and collected. He complained of an uneasy sensation about the region of the heart, numbness and partial loss of motion of the hands and feet, and great depression of strength. He had previously taken some purgative medicines; which were directed to be continued, conjoined with some doses of sulphur. In the night of January the 4th, complete paralysis of the hands and feet came on, which in the afternoon of the 5th affected all his limbs, and extended to the heart, and he died in the course of the ensuing night.

" Mr. Pearson," the writer observes, " recommends a generous diet and free exposure to cool dry air, and the patient to sit with the windows of the room open in cool weather in these cases. These were the first I ever met with of the same nature; and, as both were attended with profuse perspiration, (and if there had been no profuse perspiration,) I should think it a dangerous experiment, with these two examples before us, to expose patients under similar circumstances to cold air. If I were to meet with another similar case, I should treat it with wine, as the best cordial calculated to restore the regular action of the heart, and give sufficient doses of the solution of magnesia sulphas to diminish the effects of the mercury in the habit, and keep the patient quiet in a temperate but not cold air."

However judicious the remarks of Dr. Astbury may appear in theory, we would exhort such of our readers as may not be acquainted with the observations of Mr. Pearson on this subject, not to be guided too far by them in practice. The extensive experience of Mr. Pearson has most satisfactorily shown that wine and other cordials are nearly inefficient in this disorder, and that it is exposure to cold air on which the chief reliance is to be placed. The advantages of this practice have been most decidedly evinced at the Lock

Hospital, since it has been adopted and immediately had recourse to on the appearance of erethismus: instances of patients falling down dead on attempting to walk across the wards, in consequence of that disorder, are no longer witnessed.

---

The following additional observations on *Varioloid Diseases* to those we have already noticed, have been made by Dr. Thomson, relative to this epidemic as it appeared in the town of Lanark.—"At Mr. Owen's mills, through the obliging attention of Mr. Gibson, who has the medical charge there, I had an opportunity of seeing 118 cases of young persons affected with this disease. In its general appearances it bore a very striking resemblance to that which I have had occasion to see in Edinburgh, though, on the whole, it appeared to me to have a character considerably milder. Four only of those affected with it had previously passed through small-pox: in two of these the disease was mild, but in the other two severe. Eighty-two had this disease after having passed through the cow-pox. In a few of these it might be said to be severe; but in by far the greater number it was extremely mild, and exhibited the most convincing proofs of the efficacy of cow-pox in modifying small-pox. Thirty-two had the disease without having passed through either cow-pock or small-pox; and, what appeared to me remarkable, it had proved fatal only in one person of this class. Several, however, had been in imminent danger, and their recoveries may be tedious. Five or six of this class, as well as a considerable number of those who had previously passed through cow-pock, had the disease in a form so slight as to agree with the descriptions which have been given of chicken-pox rather than small-pox. Several individuals had experienced a severe variolous fever, without any eruption having appeared; while others had the eruption with little or no fever. The eruption itself varied in quantity, from one pustule to a number that was in some instances uncountable. By a letter which I received last evening from Mr. Gibson, I learn that the disease is still on the increase. One more instance has occurred of its having attacked a boy who had previously passed through small-pox; and one where it has attacked for the second time a lad who had previously passed through the cow-pock. In some of those who have neither undergone cow-pock nor small-pox, the disease continues, Mr. Gibson informs me, to exhibit the symptoms which have been regarded as characteristic of chicken-pox."

---

The "Medical Intelligencer" of Messrs. Burgess and Hill will be found a useful index of reference to the medical literary productions of the day, especially to such persons as may be engaged in the pursuit of any particular subject; as it will direct their attention to Memoirs, &c. respecting it, which might otherwise escape them, from their not forming distinct publications, and consequently not being comprised in the ordinary catalogues of books.

### REPORT OF DISEASES.

THE state of the inhabitants of the metropolis has been unusually healthy during the last month: the change of temperature which occurred towards the latter part of it has, however, had rather an unfavourable influence. Epidemic fever still prevails, though to less extent, and a considerable change in its character has lately taken place. Local inflammation, most frequently of the serous membranes of one of the great cavities, is almost universally evident. This is readily detected by the pain with which it is accompanied; differing in this respect from a similar affection of the mucous membranes, particularly those of the intestinal canal, which so commonly accompanies the epidemic of summer and autumn; and which is consequently so often undetected, except on dissection. It is, for the most part, accompanied with but little disturbance of the nervous system, or derangement of the functions in general, and therefore terminates without any well-marked crisis:* a careful inspection of the urine will, however, often lead to useful information in this respect.

An unusual number of deaths have occurred from apoplexy. It would be an interesting subject for investigation, to ascertain the state of the brain in those persons who die from apoplexy in this manner, when it is in some measure epidemic amongst old people, and appears to be in a great degree induced by the influence of season; but much difficulty will be experienced in carrying this to any considerable extent, in consequence of these cases occurring principally in private practice.

Inflammation of the respiratory organs, particularly of the mucous membrane of the bronchiæ, is, as usual at this season, one of the most prevalent forms of disease.

The more common chronic maladies are an aggravated state of pulmonic disorders, rheumatic inflammation, and hæmorrhoids.

A considerable change for the worse has taken place in several of the cases of disease (apparently chronic inflammation) of the heart, which come under our observation. The fate of one patient appeared to be hastened by the alteration that took place in the temperature of the atmosphere. The most urgent symptom in this instance, the subject of which was a man about thirty years of age, was an almost intolerable sense of distress about the heart, which had been increasing during the last two years, accompanied with violent agitation during sleep, and frequent interruption to it; apparently arising from great mental uneasiness. During the last few days of the patient's existence, until within a few hours of its termination, he was constantly traversing the room: no medical measures, nor voluntary efforts, could render the same position tolerable longer than a few minutes. The pulse was about 130, beating very irregularly, and with a rolling or progressive motion: the impulse of the heart could be felt over nearly the whole of the left side of the thorax. Examination of the body after death was not permitted.

---

* We use this term in the sense in which it was done by Hippocrates and Galen, (see HIPPOC. on *Affections*; and GALEN's Commentaries on the thirteenth of the second book of the *Aphorisms* of Hippocrates, and on the *Prognostics*;) not in the vague manner in which it has been employed by some modern writers. The indecision arising from this mode of applying it has caused one of the most important objects in the practice of medicine, both as regards acute and chronic diseases, to be generally disregarded.

### METEOROLOGICAL JOURNAL,

By Messrs. WILLIAM HARRIS and Co. 50, Holborn, London.

*From the 20th November to the 19th December, inclusive.*

| Day of Month. | Moon. | Rain gauge | THERM. | | | BAROM. | | De Luc's HYGROM. Dry | Damp | | Wind. | | Atmospheric Variation. | | |
|---|---|---|---|---|---|---|---|---|---|---|---|---|---|---|---|
| Nov. | | | | | | | | | | | | | | | |
| 20 | | | 41 | 46 | 39 | 29·92 | 29 81 | | 18 | 16 | SE | SE | Fog | Fine | |
| 21 | ☽ | | 41 | 43 | 39 | 29·81 | 29·74 | | 17 | 15 | SE | SE | Fog | Fine | |
| 22 | | | 40 | 41 | 48 | 29·70 | 29·69 | | 12 | 20 | SE | SE | Clo. | | |
| 23 | | | 49 | 53 | 50 | 29·70 | 29·73 | | 25 | 27 | SW | SW | Rain | Clo. | Fine |
| 24 | | ,15 | 52 | 54 | 49 | 29·75 | 29·98 | | 23 | 25 | SW | SW | Rain | Clo. | Fine |
| 25 | | ,21 | 50 | 53 | 49 | 29·98 | 30·16 | | 26 | 24 | SW | SW | Clo. | | Fine |
| 26 | | ,04 | 52 | 54 | 50 | 30·19 | 30·27 | | 23 | 27 | SW | SW | Rain | | Clo. |
| 27 | | ,19 | 51 | 54 | 52 | 30·35 | 30·39 | | 25 | 25 | WSW | SW | Clo. | | Rain |
| 28 | ◉ | ,13 | 53 | 55 | 53 | 30·43 | 30·39 | | 25 | 24 | WSW | SW | Clo. | | Rain |
| 29 | | ,15 | 56 | 60 | 50 | 30·29 | 30·23 | | 20 | 21 | W | SW | Fine | Clo. | |
| 30 | | ,12 | 53 | 59 | 50 | 30·19 | 30·13 | | 20 | 25 | WSW | W | Clo. | | Rain |
| Dec. | | | | | | | | | | | | | | | |
| 1 | | | 50 | 48 | 44 | 30·02 | 29·94 | | 16 | 18 | SW | SW | Clo. | | Fine |
| 2 | | | 45 | 47 | 43 | 30·00 | 29·85 | | 18 | 15 | WSW | W | Clo. | | Fine |
| 3 | | | 45 | 49 | 44 | 29·65 | 29·66 | | 19 | 16 | S | SSE | Rain | | Clo. |
| 4 | ☾ | ,13 | 45 | 51 | 44 | 29·60 | 29·58 | | 21 | 20 | SE | S | Rain | Clo. | |
| 5 | | ,11 | 45 | 50 | 46 | 29·51 | 29·62 | | 10 | 11 | SW | SSE | Fine | Clo. | |
| 6 | | ,07 | 49 | 50 | 45 | 29·60 | 29·51 | | 13 | 17 | S va. | S va. | Fine | | Rain |
| 7 | | ,10 | 48 | 51 | 48 | 29·44 | 29 55 | | 19 | 16 | S | SW | Clo. | | |
| 8 | | | 51 | 55 | 42 | 29·76 | 29·98 | | 20 | 21 | SSW | W | Rain | Clo. | |
| 9 | | ,21 | 45 | 49 | 40 | 30·04 | 30·07 | | 13 | 15 | NE | W | Clo. | Rain | |
| 10 | | ,20 | 42 | 45 | 38 | 30·09 | 30·12 | | 16 | 16 | W | W | Rain | | Clo. |
| 11 | | ,17 | 39 | 42 | 36 | 30·10 | 30·16 | | 12 | 16 | SW | NE | Fine | Clo. | |
| 12 | ○ | | 38 | 40 | 38 | 30·13 | 30·09 | | 10 | 9 | NE | NE | Clo. | | |
| 13 | | | 39 | 42 | 39 | 30·16 | 30·16 | | 10 | 8 | N | NE | Clo. | | Fine |
| 14 | | | 40 | 41 | 35 | 30·17 | 30·20 | | 13 | 10 | NNW | NNW | Clo. | | |
| 15 | | ,03 | 36 | 40 | 31 | 30·18 | 30·00 | | 11 | 12 | N | NE | Clo. | | Fine |
| 16 | | | 32 | 36 | 25 | 30·03 | 30·16 | | 15 | 17 | NNW | WNW | Fog | | Fine |
| 17 | | | 29 | 32 | 26 | 30·08 | 29·97 | | 20 | 23 | W | W | Fog | Clo. | Fog |
| 18 | | | 30 | 34 | 30 | 29·90 | 30·08 | | 25 | 20 | WSW | NNW | Fog | Rain | Clo. |
| 19 | | ,09 | 31 | 33 | 43 | 30·20 | 30·28 | | 21 | 20 | S | SW | Fog | Clo. | |

The quantity of rain fallen in the month of November is 1 inch and 63-100ths.

---

☞ *Our Readers will find the* List of Books in the Press, Notices of Lectures, &c. &c. *on the opposite side of the cover; where they will in future be inserted, in order that all adventitious matter may be excluded from the pages of the Journal.*

